# Study on core competencies of midwives in China

**DOI:** 10.1097/MD.0000000000034246

**Published:** 2023-10-27

**Authors:** Kejuan Sun, Jinghan Qi, Bin Luo, Lisha Yang, Chang Shu, Huijun Tang, Yanyue Qin, Yu Zang

**Affiliations:** a Department of Nursing, The First Hospital of Hebei Medical University, Shijiazhuang, China; b School of Nursing, China Medical University, Shenyang, China; c Hebei Province Key Laboratory of Nutrition and Health, Department of Clinical Nutrition, The First Hospital of Hebei Medical University, Shijiazhuang, China; d School of Nursing, Hebei Medical University, Shijiazhuang, China.

**Keywords:** core competency, midwife, midwifery education, mixed-method study

## Abstract

The aim of this study is to investigate the core competencies of midwives in China. Combination of qualitative research and quantitative research. A total of 100 midwives in 3 tertiary (Grade 3) hospitals in Shijiazhuang were investigated by using the Midwife Core Competency Scale, and simultaneously followed by semi-structured interviews with 12 midwives. The questionnaire survey showed that the average score of core competencies of midwives was 4.17 ± 0.17. The scores of midwives’ competency for labor and delivery care (4.31 ± 0.09), new-born care (4.29 ± 0.04), and postpartum care (4.25 ± 0.13) were relatively high, while the prepregnancy care had the lowest score (3.88 ± 0.07). The interview results showed that the self-perception of core midwifery competencies was not bad, the limitations of midwives’ work scope affect the core competencies, and midwifery education needs to strengthen the humanistic care and the training of obstetric knowledge and technology.

## 1. Introduction

The core competency of midwives refers to the combination of knowledge, professional and specialist skills required to perform midwifery roles in the context of midwifery education and practice^[1]^ and is an important comprehensive indicator of midwifery competence and an important basis for the development of midwifery education and regulation of midwifery practice. The International Confederation of Midwives publishes core competency indicators for midwives,^[2]^ which are constantly updated and can strongly ensure the autonomy of midwives and promote the development of the midwifery profession.^[3]^ In China, with the adjustment of fertility policy, the proportion of elderly and high-risk mothers has increased,^[4]^ thus putting forward higher requirements for midwives’ core competencies. This study aims to investigate the current situation of core competencies of midwives in Shijiazhuang city and analyze the main problems, in order to provide a theoretical basis for the training objectives and curriculum of midwifery education.

## 2. Methods

### 2.1. Study population

A mixed research method with an explanatory sequential design was used to conduct a questionnaire survey and semi-structured interviews with midwives who met the inclusion criteria. Questionnaires were first administered to midwives in 3 tertiary hospitals in Shijiazhuang from June to September 2021 using a convenience sampling method. The inclusion criteria for midwives were: working midwives who had obtained the maternal and child health technical examination certificate and nurse practice qualification certificate; working in the labor ward for more than 1 year; and voluntary participation in this study. A purposive sampling method was then used among the study participants who received the questionnaire survey, and midwives who had worked in the maternity ward for more than 5 years and maternity ward matrons were selected for semi-structured interviews from November to December 2021. All processes in this cohort study were reviewed and approved by The First Hospital of Hebei Medical University Ethics Committee.

### 2.2. Research methods

#### 2.2.1. Questionnaire.

The questionnaire consisted of 2 parts: general information, including 15 entries on age, marital status, highest education, establishment, major studied in the highest education, years of midwifery work, title, participation in teaching, time spent in midwifery training after graduation, and whether they received continuing training; midwife core competency scale, using the midwife core competency scale developed by Wang et al,^[5]^ that includes 6 dimensions of public health care, preconception care, pregnancy care, delivery care, postpartum care, and neonatal care, with a total of 54 entries, and is scored on a 5-point Likert scale, with 1 point for no competence and 5 points for full competence. The Cronbach alpha coefficient of the scale was 0.950 and the content validity was 0.95. 105 questionnaires were distributed and 100 valid questionnaires were returned, with a valid return rate of 95.24%. The data were imported into the Chinese version of EXCLE software for statistical analysis.

#### 2.2.2. Semi-structured interviews.

A total of 9 midwives and 3 maternity ward head nurses were interviewed. The interview outline was constructed in the following steps: firstly, the interview questions were initially drafted based on the literature review and the content of the scale, then the research team members discussed and revised the interview questions, and finally, 2 midwives were pre-interviewed to further refine the interview questions to determine the final version of the interview outline. The interview outline included: What are the main responsibilities of midwives in your department; What are the skills that you think midwives hold above average; In which areas do you think midwives are less competent and what are the main reasons for this; Which competencies do you think should be focused on during the education of midwifery students; and Which specialist courses do you think should be included in the midwifery curriculum. Coding was carried out using the qualitative analysis software MAXQDA 2020. Face-to-face interviews were conducted between the interviewer and the interviewee in a room with a quiet and undisturbed environment. Content analysis^[6]^ was adopted to analyze the interview data. In the first step, the 2 researchers listened to the audio recordings and transcribed them, reading all transcripts repeatedly to enhance their in-depth understanding of the data. In the second step, the 2 researchers independently reread each text carefully, highlighting meaningful phrases and writing a key word or phrase in the midwife’s words. In the third step, after coding the 3 texts, the remaining texts were coded using the codes that already existed. If the existing codes were not appropriate, new codes were added. In the fourth step, after coding all the texts, the 2 researchers cross-checked the codes and discussed any inconsistencies in the results to reach agreement. Fifthly, similar codes were grouped to identify emerging subthemes and themes. Themes were defined according to what they represented and some representative extracts were provided to illustrate these themes.

## 3. Results

### 3.1. Demographics

A total of 100 midwives were surveyed in this study, 82 (82.0%) with a bachelor’s degree or higher, 66 (66.0%) with a major in nursing, 34 (34.0%) with a major in midwifery, and 72 (72.0%) with a junior title, as detailed in Table 1.

**Table 1:** Participant demographics.

General information	N (%)
Marital status	
Unmarried	34 (34.0)
Married	66 (66.0)
Divorced	0 (0.0)
Widowed	0 (0.0)
Fertility status	
Not pregnant	42 (42.0)
Was pregnant	58 (58.0)
Highest qualification	
Secondary	0 (0.0)
College	18 (18.0)
Bachelor’s degree	82 (82.0)
Master’s degree and above	0 (0.0)
Highest degree studied	
Midwifery	34 (34.0)
Nursing	66 (66.0)
Employment	
Institutional	18 (18.0)
On contract	73 (73.0)
Others	9 (9.0)
Position	
Midwife	76 (76.0)
Group leader	8 (8.0)
Head nurse	2 (2.0)
Others	14 (14.0)
Title	
Nurse	26 (26.0)
Nurse practitioner	46 (46.0)
Supervising nurse practitioner	25 (25.0)
Associate chief nurse practitioner or higher	3 (3.0)
Type of hospital	
Specialist	56 (56.0)
General	44 (44.0)
Involved in teaching	
Yes	60 (60.0)
No	40 (40.0)
Time spent in midwifery training after graduation	
None	15 (15.0)
1–3 months	26 (26.0)
4–6 months	16 (16.0)
7 months to 1 year	13 (13.0)
More than 1 year	30 (30.0)
Continuing education received	
Yes	86 (86.0)
No	14 (14.0)

### 3.2. Core competency scores for midwives

The results of the questionnaire revealed an overall mean score of (4.17 ± 0.17) for the core competencies of midwives. The dimensions scored from highest to lowest were health care during labor, neonatal care, postnatal care, public health care, pregnancy care and preconception care (see Table 2 for details).

**Table 2:** Core competency scores for midwives (N = 100).

Dimensional entry	Mean score
Preconception care	3.88 ± 0.07
Healthcare during pregnancy	4.14 ± 013
Healthcare during delivery	4.31 ± 0.09
Postnatal care	4.25 ± 0.13
New-born care	4.29 ± 0.04
Public health care	4.15 ± 0.11
Total mean score	4.17 ± 0.17

### 3.3. Themes

From the analysis of the interview data, 4 themes were identified: self-perception of midwives’ core competence is fair, the limitations of the midwives’ work scope affect the level of core competence, midwifery education needs to emphasize on humanistic care development, and midwifery education needs to strengthen theoretical knowledge and operational skills training in obstetrics.

#### 3.3.1. Theme 1: Self-perception of midwives’ core competencies is fair.

N1: “I feel that the midwives in our department are fully competent in the care of women during labor, because our daily work is focused on the care of women during labor, followed by postnatal care and neonatal care.” N3: “We are more familiar with labor-related issues, such as labor observation, fetal heart monitoring, delivery and laceration stitching, etc.” N11: “(Core competency level) The overall level of competency is good. The ability to independently deliver a baby is not a problem, and one is capable of solving this risk independently.”

#### 3.3.2. Theme 2: The limitations of midwives’ scope of work affect core competencies.

According to the definition of the scope of work of midwives in Chinese medical institutions, midwives are mainly responsible for observing and handling normal labor and assisting obstetricians in handling abnormal labor. The scope of work of midwives focuses on health care during labor. However, midwives lack a more systematic and in-depth understanding of preconception health care, pregnancy health care and public health care. Therefore, most respondents believe that the core competencies of midwives in preconception care, pregnancy care, and public health care dimensions were relatively low. N3: “Preconception care is mainly that pregnant women will regularly go to outpatient maternity clinics, and this is mainly the responsibility of outpatient obstetricians and nurses, while midwives mainly focus on this stage of labor. So, we are not particularly familiar with pregnancy care.” N10: “preconception care is equivalent to eugenics, and I feel that this is more prenatal and relatively less covered by midwives.” N6: “This aspect of neonatal care is mainly done in the hospital by the pediatrician, and after the mother has delivered in the labor ward, we will observe her for 2 hours.” N10: “We have even less contact with public health care, perhaps more with gynecology.”

#### 3.3.3. Theme 3: Midwifery education needs to emphasize on the development of humanistic care skills.

In the training process of midwives, special attention should be paid to the cultivation of humanistic care skills for midwifery students, including empathy, love, and communication skills. N11: “When training midwifery students, it is important to develop their empathy, which are fundamental yet crucial qualities.” In addition, the interviewees pointed out that communication with mothers and families has become an important factor affecting the work of midwives, and suggested that courses on nurse-patient communication be established in the curriculum to train students’ communication skills. N5: “Many mothers are giving birth for the first time, so they will ask many questions and need professional answers and good communication from midwives to relieve their tension.” N6: “Communication is a big issue, there are many uncertainties and critical situations during labor and you have to communicate well with the family, otherwise it is very easy to create conflicts between the doctor and the patient.”

#### 3.3.4. Theme 4: Midwifery education needs to strengthen theoretical knowledge and operational skills training in obstetrics.

Theoretical knowledge and clinical skills of midwives have a direct impact on the lives of mothers and babies, so midwifery students need to strengthen their basic knowledge and technical practice. N9: “Midwives do not know much about the theoretical knowledge of preconception and pregnancy care, so if the core competencies are to be met, courses on this should be added to school education”. If the core competencies are to be met, there should be an increase in the number of relevant courses in schools. Respondents suggested that midwifery education should have a greater proportion of practical midwifery training courses, and that the content should include midwifery techniques commonly used in clinical midwifery practice. N4: “Techniques such as vaginal examination, episiotomy and suturing, neonatal resuscitation and basic early care are all commonly used in clinical practice, and I think they should be included in the practical training courses if conditions permit.”

## 4. Discussion

In this study, the total mean midwife core competency score of the respondents was (4.17 ± 0.17), which was similar to and at a good level with the core competency results of (4.08 ± 0.78) by He et al,^[7]^ (3.96 ± 0.54) by Dai et al^[8]^ and (4.18 ± 0.42) by Li et al.^[9]^ The midwives’ core competency scores for each dimension from highest to lowest were health care during labor, neonatal care, postnatal care, pregnancy care, public health care, and preconception care. This is similar to the findings of Wang,^[10]^ He,^[7]^ Wu,^[11]^ and Dai.^[8]^ The reason for the low scores on the dimensions of maternal health, public health care, and preconception health care can be related to the fact that the scope of midwifery work is mainly focused on healthcare during delivery, neonatal health care, and postnatal health care, while maternal health care, public health care and pregnancy health care are less involved. This issue continues to be a problem in the practice training of midwifery students. In the Australian placement setting for undergraduate midwifery students, care time is divided into 100 hours of antenatal examination, 40 hours of labor and delivery accompaniment and 100 hours of postnatal care,^[12]^ with placement time being allocated a greater proportion of time in the antenatal and postnatal periods. Thus, the emphasis of the midwife’s scope of work towards a few dimensions has limitations and should enhance the knowledge of clinical midwives as well as midwifery students in the areas of pregnancy care, public health care and preconception care.^[13]^

The results of the semi-structured interview were extracted and showed that the current training of midwives needs to strengthen the development of humanistic care skills and communication skills. In midwifery, midwives need to pay close attention to the emotions of the mother in labor and to reassure the family, which poses a major challenge to their humanistic and communication skills. This is in line with the philosophy of the “Humanistic Midwifery” course in Canadian and New Zealand undergraduate midwifery education, which both aim to strengthen the humanistic care of midwifery students in maternity care.^[14]^ A professional information support training system, supported by a communication skills course, can be used to improve the communication skills of midwifery students.^[15]^ Midwifery education should strengthen theoretical knowledge and operational skills practice and assessment, and professional attitude development. Midwives need to work independently to deliver babies and cooperate with doctors to deal with various emergencies during labor, which requires a strong theoretical foundation and operational skills.^[16]^ In the training process of midwifery students, theoretical knowledge and operational skills are the basis of education and should be combined with clinical skills to develop a reasonable training plan.^[17]^ Studies have been conducted to show that by educating midwifery students on professional ideology, values, career scenario guidance and emotion management, the school-institute combined education unit has improved midwifery students’ career attitudes and cultivated more positive and stable career attitudes,^[18]^ which presents a potential option for the education of modern undergraduate midwifery career attitudes.

## 5. Conclusion

In this study, the current level of core competencies of midwives in 3 tertiary hospitals in Shijiazhuang was investigated, and the results showed that the current level of core competencies of midwives is good and the training needs of midwifery profession are high, especially the training of humanistic care and communication skills. In the future, the training content, methods, assessment indicators and assessment methods of midwifery can be further explored in order to continuously improve the talent training system of midwifery.

## Acknowledgments

We are grateful to the participants to be a part of the study. We also thank Xue, PhD, for her constructive suggestions.

## Author contributions

**Conceptualization:** Yu Zang.

**Data curation:** Kejuan Sun, Yu Zang.

**Formal analysis:** Kejuan Sun, Jinghan Qi, Lisha Yang.

**Funding acquisition:** Yu Zang.

**Investigation:** Bin Luo, Chang Shu, Huijun Tang, Yanyue Qin.

**Methodology:** Yu Zang.

**Project administration:** Yu Zang.

**Supervision:** Kejuan Sun, Yu Zang.

**Writing – original draft:** Kejuan Sun.

**Writing – review & editing:** Kejuan Sun, Yu Zang, Jinghan Qi, Lisha Yang, Bin Luo, Cahng Shu, Huijun Tang, Yanyue Qin.
